# Platform Governance, Institutional Distance, and Seller Trust in Cross-Border E-Commerce

**DOI:** 10.3390/bs15020183

**Published:** 2025-02-10

**Authors:** Yulu Sun, Qixing Qu

**Affiliations:** School of Information Technology & Management, University of International Business and Economics, Beijing 100029, China; sunyulu@uibe.edu.cn

**Keywords:** platform governance mechanism, institutional distance, sellers’ trust, cross-border e-commerce

## Abstract

Trust from sellers is essential for the success of cross-border e-commerce (CBEC) platforms, and effective platform governance plays a crucial role in shaping and maintaining this trust. However, institutional distance can lead to deviations in sellers’ understanding and implementation of platform governance mechanisms, posing a threat to the establishment of trust relationships. Research on the impact of platform governance mechanisms on trust from the seller’s perspective is still relatively limited. Therefore, we aim to investigate the mechanisms through which formal governance mechanisms (normative, supervisory, and reward–punishment mechanisms) and relational governance mechanisms (community building) influence seller trust, and we examine the moderating effect of institutional distance. Through empirical analysis of 391 valid questionnaires, our results show that normative mechanisms and community building enhance seller trust by reducing their perceived risks, while the supervisory mechanism and reward–punishment mechanism directly affect seller-perceived risks and platform trust. Institutional distance negatively moderates the governance effectiveness of these mechanisms. Our findings offer significant theoretical contributions by advancing the understanding of seller behavior, extending platform governance and trust theory in CBEC research field, and enriching institutional distance frameworks, while providing practical insights for platforms to optimize governance mechanisms to foster seller trust.

## 1. Introduction

With the continuous growth of global trade and the prosperous emergence of CBEC, CBEC platforms have become a crucial bridge connecting buyers and sellers worldwide and provide new opportunities and challenges for international product flow and economic cooperation. According to statistics, China has over 5000 CBEC platform enterprises and more than 200,000 domestic enterprises engaged in CBEC transactions. With the emergence of more third-party CBEC platforms, including many overseas local e-commerce platforms opening up to Chinese sellers, the competition among major CBEC platforms will intensify, providing cross-border sellers with more choices. Therefore, ensuring that CBEC platforms possess unique competitive advantages has become a vital component for the development of CBEC in various countries.

For CBEC platforms, attracting and retaining consumers and sellers is the key marketing strategy, as most platforms profit by facilitating transactions from them (Qin et al., 2022). In the emerging platform commercial model, how to manage users has become one of the main challenges in platform governance (Pan et al., 2023; W. Li et al., 2024). Specifically, platforms must retain high-quality sellers to attract more consumers (Cui et al., 2019), and improving seller trust is a primary task of platform governance. Sellers with high trustworthiness are strategic resources that support platform development. The quality, transaction willingness, and diversity of sellers have an impact on the number of consumers and the utility they receive, thereby affecting the market share of CBEC platforms. Compared to domestic e-markets, the unique characteristics of the international market, such as high transportation costs, information asymmetry, and cultural difference, result in a high level of uncertainty for CBEC, which further emphasizes the importance of trust. Therefore, as an intermediary overseeing transactions, platforms must establish robust governance mechanisms to handle potential challenges, guarantee transaction fulfillment, and resolve disputes promptly (Grewal et al., 2010).

Previous research has demonstrated that a secure and reliable environment is crucial for gaining user trust and promoting an increase in market transactions (Lu et al., 2021; Cui et al., 2019). In particular, the basic means of establishing trust is through governance mechanism design, which can be divided into the formal governance mechanism and relational governance mechanism (Grewal et al., 2010). The formal governance mechanism focuses on the establishment of norms, using contracts as a medium to constrain the behavior of buyers and sellers, and implementing a series of incentives and penalties (Grewal et al., 2010). The relational governance mechanism complements it, primarily guiding and regulating participants’ behavior through social networks (J. Wang et al., 2022). Perfect platform governance mechanisms can facilitate communication and cooperation among cooperative enterprises and minimize conflicts caused by information asymmetry (Grewal et al., 2010; Pan et al., 2023). Similarly to consumers, sellers cannot control their CBEC platform, which exposes them to risks of e-commerce fraud, such as false returns and malicious complaints (Cui et al., 2019). In addition, due to the large number of sellers and their different credibility, the opportunistic behavior of some sellers can harm the interests of other sellers, leading to issues such as the adverse selection problem (J. Wang et al., 2022; Deng et al., 2023). Therefore, for sellers, while it is important to sell more products and achieve greater value through the platform, it is even more crucial for the platform to provide effective transaction guarantees. In this study, the formal governance mechanisms are divided into the normative mechanism (pre-transaction), supervisory mechanism (during transaction), and reward–punishment mechanism (post-transaction), while the relational governance mechanisms focus on community building. We systematically examine the role of formal governance mechanisms and relational governance mechanisms in fostering seller trust on the platform.

Institutional distance plays a crucial role in sellers’ cross-border transaction activities. CBEC platform transactions are characterized by cross-regional and cross-border nature, leading to information asymmetry and institutional differences (Ahluwalia & Merhi, 2020). In the practice of international business activities, when there is a significant institutional distance between the home country and the platform’s host country, such differences in institutional consistency become markedly pronounced, causing sellers to face challenges of legitimacy deficiency and insufficient experience in using the platform (Yan et al., 2023). Additionally, institutional distance increases the risks of uncertainty and opportunistic behavior in the cooperation process, resulting in higher transaction costs and efficiency losses, weakening multinational enterprises’ attitude and motivation to take risks, and consequently threatening their confidence (Donnelly et al., 2024). For digital platforms, the liability of foreignness brought about by institutional distance is particularly prominent, making it difficult for platforms to establish direct contact with potential users in overseas target markets and hindering their embedding into user networks in foreign markets (Brouthers et al., 2018). In this context, the trust of entities within the host country in the platform becomes a key factor in determining their decision to join the platform, while the establishment of this trust is deeply influenced by the institutional environment (Dong et al., 2017). Therefore, institutional distance plays a vital role in the relationship between the governance mechanism of CBEC platforms and seller trust, serving as an indispensable element for understanding and optimizing this complex ecosystem.

Therefore, this study centers on sellers on CBEC platforms and systematically examines the roles of formal and relational governance mechanisms in fostering sellers’ trust in the platform. We classify formal governance mechanisms into normative mechanism (pre-transaction), supervisory mechanism (during transaction), and reward–punishment mechanism (post-transaction) based on the sequence of transaction, while focusing the relational governance mechanism on community building. We constructed a theoretical model, which uses perceived risk as a mediator and institutional distance as a moderator, to reveal the underlying mechanisms and boundary conditions between platform governance mechanisms and sellers’ trust in the platform.

This paper is organized as follows. Section 2 presents a comprehensive literature review, particularly those including platform governance mechanisms, trust, and institutional distance. Section 3 develops the research model and proposes our hypotheses. Section 4 describes the empirical methodology and data collection process from CBEC platform sellers. Section 5 presents the data analysis and results. Finally, we discuss the research findings and their implications for platform governance research, study limitations, and future research directions.

## 2. Literature Review

### 2.1. Platform Governance Mechanisms

As a two-sided market, CBEC platform users consist of buyers and sellers, and issues such as counterfeit and inferior products, false returns and exchanges, and malicious complaints inevitably arise during transactions (Cui et al., 2019; J. Wang et al., 2022). Due to the virtuality of the internet, victims are unable to seek immediate offline remedies, so platforms need to establish appropriate rules to protect the rights of victims. Platform governance mechanisms manage and regulate transaction activities based on reasonable institutional frameworks (Pan et al., 2023; W. Li et al., 2024). Depending on the level of enforcement, they are divided into formal governance mechanisms (namely, contractual mechanisms) and relational governance mechanisms (namely, informal governance mechanisms) (Grewal et al., 2010).

Formal governance mechanisms refer to formal regulations and management processes established in explicit contractual forms on the platform, including standards, obligations, rules, and commitments, which clearly define the responsibilities of the stakeholders involved (Grewal et al., 2010). In the CBEC platform market, the formal governance mechanisms can be divided into normative mechanism (pre-transaction), supervisory mechanism (during transaction), reward–punishment mechanism (post-transaction), according to the order of transaction. The normative mechanism refers to the regulation and requirements of participants by the CBEC platform before the transaction, which usually includes the establishment of explicit transaction rules and policies, clarification of the rights and obligations of participants, and ensuring their understanding and agreement (W. Li et al., 2024). The supervisory mechanism refers to the monitoring of the electronic market by the CBEC platform during transaction to maintain order and regulations, ensuring that participants comply with its rules (Høgevold et al., 2022). The reward–punishment mechanism refers to giving corresponding rewards or punishments to the behavior of participants after the transaction is completed to encourage them to better comply with the regulations (Z. Zhang et al., 2020).

Although formal governance mechanisms play a crucial role in restricting or even avoiding opportunistic behavior, they cannot foresee all potential unexpected events that may occur in future transactions (Huang et al., 2014; Y. Liu et al., 2023). Relational governance mechanisms, which often have latent and informal forms of existence, can encourage interactive behavior through the development of social relationships and shared norms (Huang et al., 2014) and jointly resist the variability and uncertainty of the external environment (Chung et al., 2021), complementing formal governance mechanisms (Pan et al., 2023). Relational governance mechanisms emphasize relationships based on reciprocity, cooperation, and trust, and guide the behavior of participants through social networks (Pan et al., 2023). Mainstream CBEC platforms establish online communities for sellers as a form of relational governance mechanism, such as online forums, bulletin boards, to build embedded social relationships and help reduce risks caused by opportunistic behavior (J. Wang et al., 2022). Online communities enrich the interaction forms of sellers, cultivate a sense of commitment to the community, and facilitate smooth transactions (Minerbo et al., 2021). Moreover, sellers in the community jointly formulate rules to punish those who violate the principle of reciprocity and mutual benefit, safeguarding their common interests (Deng et al., 2023; J. Wang et al., 2022). Therefore, relational governance mechanisms reduce the reliance on formal governance mechanisms such as institutions and contracts, lower governance costs, reduce the risks brought by opportunistic behaviors, and ensure the orderly development of platform commercial activities.

Formal and relational governance mechanisms were demonstrated to be beneficial for maintaining cooperation (W. Li et al., 2024; Pan et al., 2023). Trust is primarily established based on cognitive factors, which are influenced by the institutional guarantees (Lu et al., 2021). However, relevant studies concentrated on consumers’ reactions to platform governance mechanisms (Faqih, 2022), paying less attention to their impact on maintaining a positive relationship between CBEC platforms and sellers. This is the research gap we aim to fill with this paper.

### 2.2. Trust

Online trust is one of the research topics that has received continuous attention. Many scholars and practitioners in the CBEC industry believe that the key to maintaining the healthy development of CBEC lies in gaining the trust of traders (Chen et al., 2022). Due to severe information asymmetry, online transactions are highly uncertain and risky, making trust the foundation of successful online transactions and relationship formation (Mou et al., 2020). Given the importance of trust in human economic and social activities, scholars from various fields have conducted in-depth research on the concept of trust from their respective perspectives. Mayer et al. (1995) proposed that trust is an attitude of traders towards the desired expectations of trustees in risky online environments, which involves trustees not exposing the weaknesses of the trustors, as well as their benevolence, ability, and integrity. This definition has been widely accepted by subsequent scholars. Therefore, in this study, seller trust in CBEC platforms refers to their subjective willingness to place themselves in a vulnerable state, based on their expectations of specific behaviors of the platform, including its benevolence, ability, and integrity, in an online context where the risk of uncertainty exists.

E-commerce is a virtual trading environment based on the Internet, where traders are usually strangers without interpersonal relationships. Different from traditional trading styles, the decision-making of buyers and sellers is based on online digital information rather than on-site observations and experiences, making it more difficult to establish trust in traditional interpersonal relationships in the online environment. Therefore, the trust of traders relies more on well-established institutional arrangements such as platform norms, guarantees, and commitments to protect their interests (Lu et al., 2021). Zucker (1986) pointed out that institutions are fundamental to the establishment of trust, which are important mode of trust building in non-personal competitive environments. In economics, institutional trust is generally divided into formal institutional trust and informal institutional trust (Fasli, 2007). The institutional mechanisms of e-commerce create a low-risk environment for online transactions and reduce uncertainty through clear regulatory safeguards (Sahasranamam & Nandakumar, 2020). The trust of traders in the platform largely depends on their perception of the institutional structure (Lu et al., 2021; Guo et al., 2021).

In summary, although most previous studies have focused on consumers’ views of e-commerce and developed practical solutions to enhance their engagement confidence (Faqih, 2022), the success of e-commerce also requires trust by sellers (Cui et al., 2019; Qin et al., 2022). Wei et al. (2019) found that compared to buyers, platform institutions are less protective for sellers, and the high switching costs of attracting new buyers and building online reputation make it difficult for sellers to switch. Therefore, evaluating intermediaries is more important for sellers when deciding whether to operate on a specific platform. However, existing e-commerce research is limited in explaining seller trust. Therefore, this study investigates governance mechanisms that can promote seller trust in CBEC platforms.

### 2.3. Institutional Distance

Institutional distance is used to capture the differences in the institutional environment and quality between two countries (Simonin, 1997). These differences stem from the relatively stable rules and regulations established by countries over the course of their long-term historical development, based on factors such as their unique cultural heritage, economic conditions, and geographical location. These institutions serve as important guidelines for maintaining market transaction order (Dong et al., 2017). When seller enterprises engage in international business, e-commerce reduces transaction costs associated with physical distance but also increases unique costs related to institutions, languages, and cultures. Due to natural differences among different regions, the rules and regulations, as well as administrative enforcement, vary across countries (Ahluwalia & Merhi, 2020), leading to institutional distance in CBEC transactions. Institutional distance not only profoundly affects the design and implementation of governance mechanisms on CBEC platforms but also exerts complex and far-reaching impacts on the cooperative relationship between sellers and platforms by increasing transaction costs, risks, and uncertainties (Donnelly et al., 2024).

Due to the mismatch in inherent characteristics between the platform’s host country and the home country, institutional distance may also hinder sellers from connecting themselves with the platform. The greater the differences in the institutional environments between countries, the more complex the environment faced by multinational corporations becomes (Yan et al., 2023). When significant differences in values and cognitions lead to large disparities in the institutional environments between the platform’s host country and the home country, changes in the platform’s rules, regulations, and behaviors cannot be easily perceived and understood through online communication. This results in their communication barriers and increases the difficulty for sellers to obtain information (Demirbag et al., 2007), reducing the probability of sellers acquiring the necessary information. Sellers find it challenging to grasp and adapt to the policies and regulations of the platform’s host country in the short term. This increases the complexity of the transaction environment, amplifies information asymmetry, reduces the predictability of transaction procedures and outcomes, elevates the implementation difficulty of cross-border transactions, and generates higher perceived risks, leading to the “liability of foreignness” (Dikova et al., 2010). In such cases, sellers’ sense of social identity and their perception of the platform’s responsiveness decline, which is detrimental to forming a close contractual relationship.

However, little is known about the specific role of the external institutional environment in the platform governance mechanism and trust. Existing research on institutional distance primarily focuses on its impact on multinational operations, cross-border mergers and acquisitions, international cooperation, and overseas R&D (Dong et al., 2017; Yan et al., 2023; Donnelly et al., 2024). Based on existing research that explores the influence of institutional distance on sellers’ utilization of CBEC platforms, this study theoretically analyzes its moderating effect on the relationship between platform governance mechanisms and seller trust, thereby further enriching the research framework on institutional distance.

## 3. Hypotheses Development

### 3.1. Formal Governance Mechanisms and Perceived Risk

The formal governance mechanism is regarded as a structural arrangement with legal nature used to regulate the behavior of partners in inter-firm relationships (Cravens et al., 2004). Under such constraints, the interests of all parties are more likely to be protected, information asymmetry can be effectively reduced, and opportunistic behavior can be curbed, leading to a decrease in perceived risks during the transaction process (Rodrigues et al., 2022). Formal contracts can be clearly established before transactions, observed during transactions, and verified after transactions, thereby achieving the goal of reducing transaction costs from pre-transaction, during transaction, and post-transaction. Following this sequence, this study divides formal governance mechanisms into normative mechanism, supervisory mechanism, and reward–punishment mechanism.

The institutional norms are indispensable for traders, as they can prevent information asymmetry, reduce misunderstandings, and disputes through clear transaction rules and contracts that describe the seller’s responsibilities, roles, and particular goals in detail (W. Li et al., 2024). Traders can effectively assess the penalties they will incur for violations and form a mindset based on platform ecosystem (Høgevold et al., 2022). This will gradually make participants aware of their high integration with the platform and their mutual stake, thereby reducing uncertainty and cooperative risks (Z. Li & Pénard, 2014; Rodrigues et al., 2022). Scholars have pointed out that setting high entry barriers for sellers and preventing violations due to sellers’ lack of understanding of platform rules can screen high-quality sellers and reduce the occurrence of misconduct (Yamamoto & Ohshima, 2017). This significantly increases the speculative costs for sellers, which, to some extent, discourages low-quality sellers from entering the platform. Therefore, the implementation of normative mechanism mitigates unhealthy competition and reduces sellers’ perceived risks in the transaction environment.

**H1a.**  *The normative mechanism of CBEC platforms negatively influences sellers’ perceived risks*.

Furthermore, the supervisory mechanism means the platform’s market regulation to ensure rules are abided by participants, thereby enhancing the ability to detect their opportunistic behavior (Høgevold et al., 2022). Supervision strengthens constraints on the commercial behavior of sellers, reducing friction and conflicts among collaborators (W. Li et al., 2024). Due to the potential harm that opportunistic behavior of individual sellers may cause to other sellers, especially in situations where the quality of products cannot be known in advance, adverse selection often occurs, causing the market to be dominated by opportunistic sellers, while high-quality sellers are unable to obtain price premiums (J. Wang et al., 2022; Deng et al., 2023). Therefore, through the effective implementation of the supervisory mechanism, fraudulent behavior and violations can be reduced, maintaining the fairness of transactions and reducing the perceived risks faced by sellers due to inappropriate behavior by other participants, such as negative spillover effects like platform reputation decline (Høgevold et al., 2022; Rodrigues et al., 2022).

**H1b.**  *The supervisory mechanism of CBEC platforms negatively influences sellers’ perceived risks*.

The reward–punishment mechanism, through positive incentives and negative constraints, strengthens the platform’s policies and standards (Z. Zhang et al., 2020), inspires sellers to voluntarily comply with rules and maintain a high level of relationship satisfaction, and enhances overall transaction fairness (Y. Liu & Gao, 2023) while minimizing the risks of cross-border trade sustainability. On the one hand, reward mechanisms, such as prioritized exposure and increased visibility, provide sellers with a sense of achievement and motivation to perform well, making them feel recognized for their efforts and increasing their chances of success (Hui et al., 2023). This helps to mitigate moral hazard, enhance sellers’ engagement and confidence, and ultimately create a mutually beneficial outcome for both the platform and the seller enterprises. On the other hand, the platform enforces rules by punishing violators, such as lowering search rankings and restricting trading privileges (Ma et al., 2022). When sellers observe that the platform maintains a fair attitude by enforcing constraints and punishments impartially, regardless of seller size or contribution to the platform, it helps ensure that sellers enjoy a fair competitive environment and reduces sellers’ concerns and uncertainties (Kumar et al., 1995). Punishments increase opportunistic costs for those being penalized, reduce opportunistic behavior resulting from short-term focus on immediate gains, and demonstrate the risks of opportunism to others (Zheng et al., 2019; W. Li et al., 2024). Additionally, buyers’ dishonest behaviors, such as false returns and malicious complaints, cause economic losses and reputation risks for sellers (Yamamoto & Ohshima, 2017). However, if the platform can take effective penalties to combat such behaviors, such as account suspension or restricted purchasing abilities, it can instill confidence in sellers and reduce their concerns about buyer opportunistic behavior (Cui et al., 2019; San-Martín & Jimenez, 2017). In conclusion, the punishment mechanism has a deterrent effect and can reduce the damage and risks caused by behavioral uncertainties (Y. Liu & Gao, 2023). Therefore, we hypothesize as follows:

**H1c.**  *The reward–punishment mechanism of CBEC platforms negatively influences sellers’ perceived risks*.

### 3.2. Relational Governance Mechanism and Perceived Risk

The trust-based informal governance mechanisms, also known as relational governance mechanisms, establish shared values and non-legal regulations through relationship norms (Chung et al., 2021; W. Li et al., 2024). Many e-commerce platforms intentionally build online communities as their organizational form (Minerbo et al., 2021). Online communities establish their social norms, serving as a powerful interactive medium influencing participants’ behavior (J. Wang et al., 2022). Therefore, platforms often entrust community management to their members, while focusing on providing technical assistance. In online communities, information and knowledge exchange quickly weakens members’ perception of transaction risk, and sellers are more likely to observe opportunistic behavior and respond quickly (Shen et al., 2022). Close membership in the community allows sellers to monitor and punish violators (J. Wang et al., 2022). In summary, effective peer monitoring within a community exerts pressure on members to adhere to social norms, helping to reduce the occurrence of misconduct and reduce the risk of negative impact on other sellers’ reputation due to individuals’ opportunistic behavior (Deng et al., 2023). Additionally, through interactions with the community, sellers can establish connections and provide mutual assistance with other members, facilitating emotional communication and support among community members (Shen et al., 2022). When facing challenges, sellers can receive encouragement, support, and understanding from community members, indirectly helping them overcome difficulties with a positive attitude (J. Wang et al., 2022), which suppresses their perception of risk. Therefore, we hypothesize as follows:

**H2.**  *The community building of CBEC platforms negatively influences sellers’ perceived risks*.

### 3.3. Perceived Risk and Platform Trust

The perceived risk describes the uncertainty that sellers have regarding the potential negative outcomes of conducting transactions on CBEC platforms (Ma et al., 2022). Various potential uncertainties and risks, such as transaction security, cultural differences, payment, law, and intellectual property protection, are considered negative factors that hinder physical and online transactions (Chen et al., 2022; A. Liu et al., 2021), which can significantly impact the public’s confidence in their decision-making. Therefore, risk is a necessary factor for trust to exist. From a risk decision perspective, trust is essentially a rational judgment made in a risk environment, and it is ultimately manifested by the acceptance of risk (Kim et al., 2008). Mayer et al. (1995) argued that trustors are willing to take risks and choose to trust only when their level of trust exceeds their perceived risk threshold. Recent research has shown that, in an online environment, risk perception has a negative impact on multidimensional trust (Mou et al., 2020). When trustors’ risk perception of the trustee decreases, they reduce their worries and fears, thus becoming more tolerant of risk. Perceived risk acts as a strong deterrent in the formation of trust, hindering people’s adoption of internet-based transaction (Kadi & Amalia, 2021). For sellers, if they perceive a high level of risk on the platform, such as consumer fraud, unfair competition, and potential logistic losses, they may doubt the platform’s integrity and reliability (Cui et al., 2019). Conversely, if the platform can effectively manage risks and provide reasonable support and protection to sellers, their trust will be enhanced. Therefore, we hypothesize as follows:

**H3.**  *Perceived risk negatively influences sellers’ platform trust*.

### 3.4. The Mediating Role of Perceived Risk

Previous research has shown that a secure trading environment is crucial for gaining user trust and increasing market transaction volume (Yamamoto & Ohshima, 2017; Guo et al., 2021). Meanwhile, the fundamental means to achieve trust is through the design of governance mechanisms. Based on institutional trust theory, when traders perceive control in the external environment, they can recognize that the platform, by leveraging relevant institutional controls, can, to some extent, guarantee the security of transactions, reduce the perceived risks and uncertainties of participants, and thus tend to trust (Zucker, 1986; Wei et al., 2019; Lu et al., 2021). Firstly, normative mechanism provides sellers with clear rules and standards, enabling them to understand the platform’s expectations and avoid the risks associated with non-compliance (W. Li et al., 2024; Rodrigues et al., 2022). Secondly, supervisory mechanism can promptly identify and correct non-compliant behavior by monitoring and inspecting traders’ actions (Høgevold et al., 2022). By handling disputes between sellers and consumers in a fair and transparent manner, the platform conveys its commitment to maintaining fair transactions, reducing sellers’ risk perceptions during the transaction process (Rodrigues et al., 2022). Finally, the reward–punishment mechanism sends a clear signal to sellers that the platform takes rule enforcement seriously (Z. Zhang et al., 2020). By providing reward incentives, sellers are encouraged to comply with rules and exhibit good behavior, thereby reducing their perceived operational risks (Hui et al., 2023) and increasing trust in the platform. Implementing strict penalties allows sellers to perceive the platform’s zero-tolerance attitude towards non-compliant behavior, reducing the risk of compliant sellers being implicated (Ma et al., 2022). In conclusion, a well-designed formal governance mechanism of the platform can decrease the implementation of opportunistic behaviors by participants (Sahasranamam & Nandakumar, 2020), thus reducing the risk perception of platform users and giving them more confidence to operate on the platform (Rodrigues et al., 2022). Therefore, we hypothesize as follows:

**H4.**  *Perceived risk mediates the relationship between formal governance mechanisms of CBEC platforms and sellers’ platform trust*.

**H4a.**  *Perceived risk mediates the relationship between normative mechanisms of CBEC platforms and sellers’ platform trust*.

**H4b.**  *Perceived risk mediates the relationship between supervisory mechanisms of CBEC platforms and sellers’ platform trust*.

**H4c.**  *Perceived risk mediates the relationship between reward–punishment mechanisms of CBEC platforms and sellers’ platform trust*.

The relational governance mechanisms of a platform can help sellers break free from the shackles of short-term interests and enhance interaction among community members to improve their understanding of the market and platform, which further weakens sellers’ perception of risk and cultivates their trust towards the platform (Chung et al., 2021; W. Li et al., 2024). Based on shared values and social norms, community building establishes confidence among members and clarifies their relationship expectations. This means that conflicts and problems within the community will be resolved, and no member will engage in opportunistic behavior, thereby reducing sellers’ concerns about opportunistic behavior and uncertainty (J. Wang et al., 2022). At the same time, community building can promote cooperative communication, and valuable information provided by others can directly assist sellers in overcoming difficulties and making correct decisions, thus better avoiding risks and building confidence (Shen et al., 2022). In addition, community building strengthens the cohesion of the network, and when sellers receive care from similar peers, they can release and stabilize their emotions (Shen et al., 2022). This common experience and emotional connection can make sellers feel comfortable and belong, reduce their perception of risk, and further motivate them to overcome challenges (J. Wang et al., 2022). In conclusion, community building can positively influence members’ confidence in the platform by reducing their perception of risk. Therefore, we hypothesize as follows:

**H5.**  *Perceived risk mediates the relationship between community building of CBEC platforms and sellers’ platform trust*.

### 3.5. The Moderating Role of Institutional Distance

This study employs institutional distance to capture the institutional environment differences in which CBEC platforms and sellers operate (Simonin, 1997). Based on the liability of foreignness theory, when institutional distance is significant, sellers, due to their unfamiliarity with the laws, regulations, business practices, and cultural values of the target market, may experience greater uncertainty and doubts about the platform’s governance mechanisms (Boehe, 2011; Chao & Kumar, 2010; Dong et al., 2017). This uncertainty manifests as concerns regarding the fairness, enforcement, and transparency of these mechanisms. Sellers may worry about biases or unfairness in the platform’s governance mechanisms and fear that their rights and interests cannot be effectively protected, thereby increasing their perceived risk (Donnelly et al., 2024).

Institutional distance can lead to variations in sellers’ understanding of platform normative mechanisms. When the institutional distance between the platform’s host country and the sellers’ home country is significant, sellers may struggle to grasp and adapt to the policies and regulations of the host country in the short term, exacerbating information asymmetry and increasing the difficulty of implementing cross-border transactions (Dikova et al., 2010). Based on the theory of organizational legitimacy, such differences may cause sellers to misunderstand or be dissatisfied with platform regulations, perceiving certain norms as overly stringent or incompatible with their business practices, thereby heightening their perceived risk (Dong et al., 2017). Furthermore, institutional distance results in substantial discrepancies in institutional and market coherence, making it challenging for sellers to adapt due to a lack of market experience or legitimacy (Yan et al., 2023). Sellers often need to invest more time and resources to familiarize themselves with the institutional norms of the local market, and they must bear additional costs arising from implicit rules within various national institutional environments, as well as the process of establishing connections and gaining recognition from stakeholders. This further increases uncertainty and risk, intensifying the burden of information processing (Donnelly et al., 2024). Therefore, we hypothesize as follows:

**H6a.**  *Institutional distance negatively moderates the relationship between normative mechanism and sellers’ perceived risk*.

Institutional distance influences sellers’ acceptance of platform supervisory mechanism. When institutional distance is significant, sellers may develop resistance to the platform’s supervisory measures (Chao & Kumar, 2010; Chen et al., 2022). This is because sellers perceive the platform’s supervisory measures as overly strict or unreasonable, significantly differing from the supervisory approaches they are familiar with (Ghate & Nagendra, 2005). This resistance leads to an increase in sellers’ perceived risk (R. Wang & Sui, 2019). Additionally, institutional distance poses more challenges for the platform during the supervisory process, such as information asymmetry and enforcement difficulties, thereby affecting the effectiveness of the supervisory mechanism (Caixe et al., 2024). Therefore, we hypothesize as follows:

**H6b.**  *Institutional distance negatively moderates the relationship between supervisory mechanism and sellers’ perceived risk*.

Institutional distance leads to differences in sellers’ expectations and the degree of implementation of platform reward–punishment mechanism. Different countries have varying regulations regarding the severity of penalties for violations, which causes sellers to have doubts about the platform’s reward–punishment mechanism (Lacey, 2011). In some countries, platforms face difficulties in effectively punishing violations because local laws and regulations limit their disciplinary powers (Harding & Ireland, 2022). In other countries, however, platforms have broader disciplinary powers (Eriksson et al., 2017). This discrepancy results in sellers feeling a sense of unfairness or dissatisfaction with the platform’s reward–punishment mechanism, thereby increasing their perceived risk (L. Zhang et al., 2022; Chua et al., 2015). Therefore, we hypothesize as follows:

**H6c.**  *Institutional distance negatively moderates the relationship between reward–punishment mechanism and sellers’ perceived risk*.

The cultural differences among different countries and regions have shaped diverse cultural beliefs, thinking patterns, and behavioral models, giving rise to distinctive corporate cultures (Yan et al., 2023). The organic integration of multiculturalism exacerbates cultural conflicts between parties involved in cross-border transactions, leading to higher cross-cultural coordination costs (Lawrence et al., 2021). Based on the theory of empathy effect, the increase in institutional distance makes it difficult for sellers to integrate into local online communities (Donnelly et al., 2024). This is because the socio-cultural and value differences among different countries result in significant variations in the behaviors and attitudes of online communities (Dau et al., 2022). Sellers find it challenging to understand and accept the rules and values of these communities, thereby increasing their perceived risk (Yan et al., 2023; D. Li et al., 2021).

**H7.**  *Institutional distance negatively moderates the relationship between community building and sellers’ perceived risk*.

**Figure 1:**
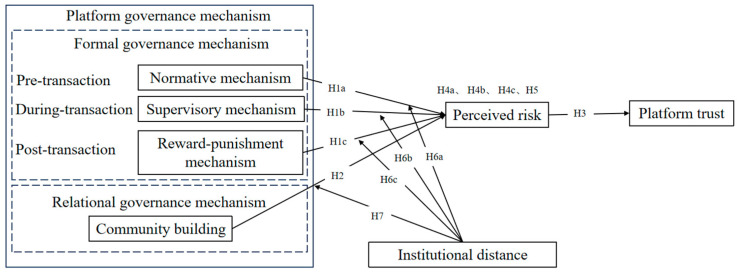
Research model.

In summary, the research hypotheses are presented in Figure 1, outlining the logical relationships among CBEC platform governance mechanisms, sellers’ perceived risk, and platform trust, while also examining the moderating effect of institutional distance. Specifically, H1a, H1b, and H1c aim to verify the impact of formal governance mechanisms on sellers’ perceived risk. H2 aims to validate the influence of relational governance mechanism on sellers’ perceived risk. H3 is designed to test the effect of sellers’ perceived risk on their trust in the platform. H4a, H4b, and H4c are intended to examine the mediating role of perceived risk in the relationship between formal governance mechanisms and sellers’ platform trust. H5 aims to investigate the mediating effect of perceived risk in the relationship between relational governance mechanism and sellers’ platform trust. H6a, H6b, and H6c seek to confirm the moderating effect of institutional distance on the relationship between formal governance mechanisms and sellers’ perceived risk. H7 aims to validate the moderating role of institutional distance in the relationship between the relational governance mechanism and sellers’ perceived risk.

## 4. Methodology

### 4.1. Sample and Data

Our questionnaire aims to investigate the operating sellers on CBEC platforms, including two parts. The first part includes basic information about sellers, such as their store opening time, identity, primary industry, employee size, trade transaction, average per transaction, and their CBEC platforms. The second part measures relevant variables, including normative mechanism, supervisory mechanism, reward–punishment mechanism, community building, perceived risk, institutional distance, and platform trust, with a total of 28 items.

Considering the reliability and extensiveness of the questionnaire, we utilized the paid targeted distribution service provided by “Credamo” (www.credamo.com) on 4 January 2024 to ensure the precise distribution to operating sellers on CBEC platforms. Moreover, a translation and back-translation method was used for mature scales derived from English literature. Firstly, the English scale was translated into Chinese, and then two experienced American researchers in the field of management were invited to back-translate the Chinese scale into English, comparing it with the original and the translated English versions. Prior to the formal distribution, a small-scale pilot test (100 samples) was conducted to revise and optimize the questionnaire design. We collected 497 questionnaires, and after excluding incomplete, over-filled, and randomly oriented invalid questionnaires, 391 valid questionnaires were obtained, with a valid response rate of 78.67%.

### 4.2. Measurement

In our research model, platform trust is the dependent variable, platform governance mechanisms are the independent variables, sellers’ perceived risk is the mediating variable, and institutional distance is the moderating variable. We drew on existing mature scales and made appropriate adjustments. All variables were measured by a Likert 7-point scale. Our specific items are presented in Table 1.

Platform governance mechanisms include the normative mechanism, supervisory mechanism, and reward–punishment mechanism of formal governance, as well as the community building of relational governance. The measurement of normative mechanism draws on the scale developed by Pan et al. (2023), describing the norms and requirements of CBEC platforms for the behavior of traders, with a total of four items. The scale for supervisory mechanism comes from Li et al. (2024) and Grewal et al. (2010), describing the supervision of electronic markets by CBEC platforms, with a total of four items. The measurement of reward–punishment mechanism refers to the scales developed by Y. Liu and Gao (2023) and D. T. Wang et al. (2013), examining the platform governance mechanisms from the perspectives of incentives and penalties, with a total of four items. The items design for community building mainly refers to the studies of Shen et al. (2022) and J. Wang et al. (2022), investigating how CBEC platforms guide and regulate participants’ behavior through social networks, with a total of four items.

To measure sellers’ perceived risk, this study extracted four items from the scales developed by Cui et al. (2019) and Wei et al. (2019), describing sellers’ perceptions of the negative impacts and uncertainties on the CBEC platform. We extracted four items from the scales of Geldes et al. (2015) and Chao and Kumar (2010) to measure institutional distance, reflecting the differences in the institutional environments between sellers and CBEC platforms. The measurement items for platform trust were derived from the scales of Qin et al. (2022) and Chen et al. (2022), assessing sellers’ confidence in the CBEC platform, with a total of four items.

To ensure the rationality and scientificity of the research results, this study selected a series of seller characteristics as control variables, including store opening time, identity, employee size, trade transaction, and average per transaction, in order to prevent these factors from interfering with our results.

## 5. Analyses and Results

### 5.1. Descriptive Statistics of Samples

The demographic characteristics of the sample are shown in Table 2. In terms of store opening time, 23.27% of the sample companies were engaged in CBEC for less than 1 year, 40.41% for 1–3 years, 25.83% for 3–5 years, and 10.49% for over 5 years. The distribution of the surveyed sellers is relatively balanced. Over 50% of the samples belong to senior and middle managers, who have relatively good understanding of the company’s cross-border activities and can provide accurate and objective evaluations of platform trust, thereby enhancing the credibility of the sample data. The primary industries include electronic products and daily necessities, which are characterized by international products, aligning with the current situation of CBEC companies in China. In terms of employee size, approximately 90% of the samples have less than 500 employees, indicating that small and medium-sized enterprises are the main participants on CBEC platforms in China. According to the statistics on store trade transactions, 69.31% of the stores have an annual transaction exceeding RMB 1 million, with the majority having an average transaction between RMB 100 and RMB 300. This reflects that cross-border transactions in China are dominated by small orders but with higher profitability. In terms of platform selection, the top three platforms used by the samples are Alibaba, Amazon, and AliExpress, with many companies choosing multiple platforms for export simultaneously.

### 5.2. Reliability and Validity

Reliability analysis is a prerequisite for ensuring the effectiveness of hypothesis testing and model fit. As shown in Table 3, all Cronbach’s alpha (CA) and composite reliability (CR) values in this study exceed the threshold of 0.7, indicating good reliability of the questionnaire.

When the measurement indicators of convergent validity reach certain values, it indicates that all measurement items of the same construct have a high level of correlation. As shown in Table 3, except for a few items (MM1, MM3, PR1, and PT2) with factor loadings below 0.7, most of the construct values are above 0.7. The AVE values of each construct exceed the threshold of 0.5, indicating that the model has high convergent validity. As shown in Table 4, the square roots of the AVE for all seven constructs are greater than their correlations with other constructs, indicating that this study has good discriminant validity.

### 5.3. Common Method Bias (CMB)

In behavioral research, particularly when using self-report questionnaires, common method bias (CMB) is a common potential issue. Specifically, it refers to the bias or correlation between data that arises from using the same method or measurement tool. Given that our data are derived from the same group of respondents, addressing the problem of CMB becomes crucial. This study primarily examines it from questionnaire design and statistical analysis.

In terms of questionnaire design, this study attempts to ensure that the questionnaire items are direct, specific, and brief, avoiding ambiguous concepts and asking. In terms of statistical analysis, we adopt Harman’s single-factor test, variance inflation factors (VIFs), and a single common method factor analysis to test for CMB. Firstly, the results of the unrotated factor analysis show that the first factor explains 24.914% of variances, which is less than 40%, and no single factor explained too much. Secondly, the VIF values below the threshold of 10, showing no severe multicollinearity problem in our research model. Finally, by adding a common method factor to our seven-factor model and conducting a confirmatory factor analysis, the results show minor differences in fit indices compared to the original model. The changes in RMSEA and SRMR are within 0.05, while the changes in CFI and TLI are within 0.1, providing evidence that there is no significant CMB issue.

### 5.4. Structural Equation Model (SEM)

SEM can simultaneously examine the relationships among multiple latent variables. Therefore, we used SEM to empirically test our hypotheses and analyze path coefficient. The model fit results are presented in Table 5, indicating a good fit of our model structure.

#### 5.4.1. Direct Effect Analysis

As shown in Table 6, the normative mechanism (β = −0.513, *p* < 0.001), supervisory mechanism (β = −0.163, *p* < 0.05), and reward–punishment mechanism (β = −0.212, *p* < 0.01) of formal governance mechanisms have significant negative effects on sellers’ perceived risk, supporting H1 (including H1a, H1b, and H1c). The community building (β = −0.843, *p* < 0.001) of relational governance mechanism has a significant negative effect on sellers’ perceived risk, supporting H2. Sellers’ perceived risk (β = −0.357, *p* < 0.001) has a significant negative effect on platform trust, supporting H3.

#### 5.4.2. Mediation Effect Analysis

Using the bootstrap test with 5000 samples and a 95% confidence level, the results of the mediation effects are shown in Table 7. The mediation effect of perceived risk between the normative mechanism and platform trust is 0.038, with the confidence interval for the indirect effect not including 0, and the confidence interval for the direct effect including 0. This indicates that perceived risk plays a significant full mediating role between the normative mechanism and platform trust, supporting H4a. The mediation effect of perceived risk between community building and platform trust is 0.064, with the confidence interval for the indirect effect and direct effect not including 0. This indicates that perceived risk plays a significant partial mediating role between community building and platform trust, supporting H5. The confidence intervals for the indirect effects of perceived risk between supervisory mechanism and platform trust, as well as between reward–punishment mechanism and platform trust, include 0. This suggests that perceived risk does not mediate between supervisory mechanism and platform trust, nor between reward–punishment mechanism and platform trust, indicating that H4b and H4c are not supported.

#### 5.4.3. Moderating Effect Analysis

As shown in Table 8, institutional distance has a significant negative moderating effect in the relationship between normative mechanism and perceived risk (interaction effect: β = 0.151, *p* < 0.001), between supervisory mechanism and perceived risk (interaction effect: β = 0.114, *p* < 0.001), between reward–punishment mechanism and perceived risk (interaction effect: β = 0.014, *p* < 0.05), and between community building and perceived risk (interaction effect: β = 0.104, *p* < 0.001). This indicates that the higher the platform big data capability, the stronger the negative correlation between the normative mechanism and perceived risk, between the supervisory mechanism and perceived risk, between the reward–punishment mechanism and perceived risk, and between community building and perceived risk, supporting H6a, H6b, H6c, and H7.

## 6. Discussion

We demonstrated the effectiveness of platform governance mechanisms on the formation of trust by sellers. The formal governance mechanisms of the platform are represented by the normative mechanism, supervisory mechanism, and reward–punishment mechanism, while community building represents the relational governance mechanism of the platform. Therefore, strengthening platform norm formulation, supervision, reward–punishment, and community building can reduce sellers’ perceived risks and increase their trust in the platform. As mentioned earlier, the CBEC platform establishes social norms and builds communities, which reduces sellers’ concerns about uncertainty risks and fosters trust. However, the supervisory mechanism and reward–punishment mechanism directly influence sellers’ platform trust, and perceived risk does not mediate this relationship. The reason may be that when sellers perceive the existence of the supervisory mechanism and the effectiveness of the reward–punishment mechanism, they may pay less attention to potential risks and focus more on the platform’s management and rule enforcement. Research has shown that even if the public perceives risks, transactions can still be sustained as long as they hold positive expectations about their behaviors (Faqih, 2022; Nguyen-Phuoc et al., 2021; Yang et al., 2015). Therefore, even if sellers are aware of potential risks in certain transactions, their trust will not be undermined by risk perception as long as they believe that the platform can effectively manage and address these risks. In an environment with robust supervisory mechanisms and clear reward–punishment systems, sellers are more concerned with how the platform handles transaction disputes, rewards quality services, and penalizes violations. These factors, which directly impact sellers’ interests, become the primary basis for their evaluation of platform trustworthiness. By contrast, the perception of transaction risks may become relatively secondary.

Moreover, this study also verifies the impact of institutional distance on the implementation effectiveness of platform governance mechanisms. The empirical results indicate that when institutional distance is high, normative mechanism, supervisory mechanism, reward–punishment mechanism, and community building have a weaker impact on seller perceived risk. This suggests that in environments with large institutional differences, the effectiveness of platform governance mechanisms may be somewhat limited.

### 6.1. Theoretical Contributions

(1) We focus on the trust relationship between the platform and sellers, one of the key stakeholders in CBEC platforms, filling the gap in the theoretical study from sellers’ perspectives. Existing studies mostly analyzed the trust of consumers in sellers and platforms (Faqih, 2022), while little research from the perspective of sellers focuses on their trust in consumers and CBEC platforms. This study constructs a theoretical model of platform trust for sellers and empirically analyzes the effect mechanism of CBEC platform governance mechanisms on platform trust, which provides insights in the field of platform governance and trust.
(2) This study divides four effective platform governance mechanisms from formal and relational perspectives into normative mechanism, supervisory mechanism, reward–punishment mechanism, and community building, which help establish close relationships between CBEC platforms and sellers. We expand the research field on platform governance mechanisms. Our result shows that the effective implementation of formal governance mechanisms, such as normative mechanism (pre-transaction), supervisory mechanism (during transaction), and reward–punishment mechanism (post-transaction), helps prevent and resist improper behaviors in transactions. These enable participants’ interests to be protected, and the perceived risk is reduced. In addition, community building of relational governance mechanism, through interaction in community, not only help sellers gain more experience and advice but also allow other members to supervise and constrain sellers’ behavior and performance, thereby reducing the risk of opportunistic behavior by individuals negatively affecting other sellers. The reduction in perceived risk reduces sellers’ concerns and fears, thereby developing trust in the platform’s integrity and reliability.
(3) This study analyzes, from an institutional perspective, how institutional factors affect the effectiveness of CBEC platform governance mechanisms, revealing the mechanism by which institutions influence sellers’ trust in the platform. Although the institutional perspective has been widely used in existing international strategic studies, which have focused on host country selection, entry modes, ownership structures, and expatriate strategies (Dong et al., 2017; Yan et al., 2023; Donnelly et al., 2024), with little attention on how institutional factors in the external environment affect platform governance effectiveness. This study, from an institutional perspective, investigates how the institutional distance between CBEC platforms and sellers affects sellers’ risk perception towards the platform governance mechanisms, enriching the theoretical research on governance from an institutional perspective and providing empirical evidence for sellers to use CBEC platforms for international expansion.

### 6.2. Managerial Implications

This study helps provide guidance for CBEC platforms to manage numerous seller users. Considering the positive impact of platform governance mechanisms on the seller-platform relationship, platform operators must understand how to design, implement, and maintain them. In terms of formal governance mechanisms, CBEC platforms should strengthen the regulation of seller behavior before transaction, establish strict supervision mechanisms during transaction, and adopt effective incentives and penalties to encourage sellers to comply with platform regulations and standards after transaction. In terms of relational governance mechanisms, platforms can encourage sellers to participate in community activities, share experiences, and support each other, forming positive social norms and enhancing cooperation among sellers. The simultaneous use of these two governance mechanisms can complement each other and bring higher benefits to the platform and its seller users. The stronger the platform governance capability, the more adept it is at facilitating the sharing of information and culture between supply and demand parties from different institutional environments, mitigating the negative impacts brought about by institutions, culture, and geography (Faraj et al., 2016). In addition, when designing and implementing governance mechanisms, CBEC platforms must fully consider the impact of institutional distance to ensure that the governance mechanisms can effectively function in different institutional environments. At the same time, platforms need to closely monitor changes in the institutional environment and promptly adjust and optimize governance mechanisms to address sellers’ perceived risks in different institutional contexts.

CBEC sellers actively participate in platform governance and online communities, effectively managing operational risks, forming a community of shared interests with the platform, and establishing an excellent trust relationship. As the initial investment and subsequent maintenance of platforms need to consume the seller’s resources, so selecting a suitable CBEC platform is crucial, which determines whether the seller can successfully enter the target market and avoid potential detours during its operation. Our findings affirm the critical role of platform governance mechanisms in fostering seller trust in the platform. Therefore, sellers should actively participate in and abide by the platform’s rules and contracts, urge the platform to provide transparent supervision mechanism and fair incentives and penalties, avoid violations, and establish a favorable commercial image. Additionally, sellers are required to maintain their social relationships, actively participate in community activities, and expand their information sources and emotional support, thereby helping them reduce the risks and transaction costs. Lastly, multinational sellers should pay close attention to the institutional quality in the platform’s host country and the institutional distance from their home country, fully recognizing the costs and risks that institutional distance may bring, including legality issues, liability of foreignness, and country-of-origin disadvantage. To this end, sellers should consciously and continuously monitor the overseas business environment, promptly identify potential risks, accumulate experience in dealing with institutional heterogeneity of overseas platforms, and enhance their problem-solving ability and sensitivity in the face of institutional logic conflicts.

### 6.3. Limitations and Future Research

We have drawn valuable conclusions and managerial implications in terms of platform governance mechanisms and seller trust in the platform. However, there are still some limitations that provide directions for future research. Firstly, our data mainly came from self-reported questionnaire surveys. Although this study did not detect common method biases, future research can adopt additional methods, such as interviews, behavioral experiments, and longitudinal studies, to complement and validate our findings. Secondly, this study only focused on empirical analysis of sellers on CBEC platforms, as we believe that platform governance for sellers is the foundation, and by enhancing seller trust, more high-quality buyers can be attracted. However, future research can further explore platform governance mechanisms towards consumers and compare and analyze the governance differences among different platforms, making the research in the field of platform governance more comprehensive. Lastly, CBEC systems are complex, and factors such as national policies, market environment, platform quality, and company capital can affect the behavior of cross-border sellers. This study mainly considers the influence of platform governance mechanisms on seller trust, but future research still needs to expand horizontally and explore the mutual influence of multiple factors from multiple perspectives in a more in-depth manner.

## 7. Conclusions

This study focuses on sellers on CBEC platforms, explores the effect mechanism of platform governance mechanisms on platform trust for sellers, and analyzes the mediating effect of perceived risk, as well as the moderating effect of institutional distance. Based on survey data from 391 sellers on CBEC platforms, our empirical analysis results reveal that the formal and relational governance mechanisms within platform have a significantly negative impact on sellers’ perceived risk, which in turn negatively affects sellers’ trust in the platform. Perceived risk plays a fully mediating role between normative mechanism and sellers’ platform trust, and a partial mediating role between community building and sellers’ platform trust, but does not mediate between supervisory mechanism and sellers’ platform trust, as well as between reward–punishment mechanism and sellers’ platform trust. Institutional distance has a significant negative moderating effect on the relationship between platform governance mechanisms (normative mechanism, supervisory mechanism, reward–punishment mechanism, community building) and seller perceived risk.

This study makes contributions in the following three aspects. Firstly, this study offers a novel perspective by examining CBEC platform trust from the sellers’ viewpoint. By investigating the formation mechanism of seller trust, we contribute to the understanding of seller behavior, enrich trust theory in the CBEC context, and broaden the scope of research in this field. Secondly, this study systematically categorizes platform governance mechanisms and evaluates their effects on sellers’ platform trust, providing valuable theoretical insights into platform governance theory. Lastly, this study explores the moderating role of institutional distance in the relationship between platform governance mechanisms and seller trust, further enriching research frameworks on institutional distance.

## Figures and Tables

**Table 1:** Variable definition and metric.

Constructs	Items
Normative mechanism	The platform has designed operating rules that ensure secure and fair transactions, such as user agreements, buyer–seller behavior norms, evaluation rules, and dispute resolution rules, which define the rights and obligations of various roles.
	The platform requires sellers to be aware of market management norms, industry management norms, and violation handling norms.
	The platform strictly audits the operational qualifications of sellers, including business licenses and operational scope.
	The platform regularly updates and improves transaction rules and policies to adapt to market and regulatory changes.
Supervisory mechanism	The platform closely monitors and supervises the behaviors of buyers and sellers that violate the transaction rules.
	The platform has established an effective complaint handling mechanism to promptly process complaints and disputes.
	When a transaction dispute occurs, the platform timely, efficiently, and fairly helped me resolve the dispute.
	The platform effectively maintains the order of transactions.
Reward–punishment mechanism	The platform rewards sellers who comply with regulations, such as providing priority display and increasing exposure opportunities.
	The platform imposes penalties on sellers who engage in violation behaviors, such as fines, sales restrictions, and credit reduction.
	The platform is able to reward or penalize sellers in a timely manner based on their behaviors.
	The platform is able to fairly reward or penalize sellers according to their behaviors.
Community building	Establishing a community among sellers is important.
	Reciprocal norms within the community facilitate smooth activities within the community.
	Members of the online community have high levels of activity participation.
	When I encounter difficulties, some other sellers who interact with me will comfort and encourage me.
Perceived risk	Selling products through this platform may incur significant costs in logistics, such as long delivery times, high shipping prices, high package loss rate, and customs clearance issues.
	Selling products through this platform may result in the leakage or misuse of my personal information.
	I am concerned about whether this platform can guarantee a fair competitive environment in the market.
	Overall, this platform makes me feel high levels of risk.
Institutional distance	The political, economic, legal systems, and business practices of the country where the platform is located are significantly different from those of our home country.
	The internal market institutions, behavioral norms, and organizational culture of the country where the platform is located are significantly different from those of our home country.
	The platform differs significantly from platforms in our home country in terms of operational style, strategic goals, and other aspects.
	The language, religion, lifestyles, and values of the country where the platform is located differ greatly from those of our home country.
Platform trust	The platform is trustworthy.
	The platform has a high level of integrity.
	Trading on this platform is safe and reliable.
	I believe that the platform can protect my interests.

**Table 2:** Sample characteristics.

Demographic Variable	Items	Frequency	Percentage
Store opening time	1 year or less	91	23.27%
	1 to 3 years	158	40.41%
	3 to 5 years	101	25.83%
	Over 5 years	41	10.49%
Identity	Owner	49	12.53%
	Senior manager	52	13.30%
	Middle manager	115	29.41%
	Junior manager	175	44.76%
Primary industry	Clothing and accessories	80	20.46%
	Home textiles	16	4.09%
	Electronic products	112	28.64%
	Daily commodities	62	15.86%
	Beauty care	21	5.37%
	Shoes and bags	20	5.12%
	Gifts and toys	17	4.35%
	Office supplies	16	4.09%
	Foodstuffs	31	7.93%
	Others	16	4.09%
Employee size	Less than 10	39	9.97%
	10 to 30	107	27.37%
	30 to 50	98	25.06%
	50 to 100	72	18.41%
	100 to 300	33	8.44%
	300 to 500	42	10.74%
Trade transaction (RMB)	Below 10,000	3	0.77%
	10,000 to 100,000	19	4.86%
	100,000 to 500,000	49	12.53%
	500,000 to 1 million	49	12.53%
	1 million to 5 million	92	23.53%
	5 million to 10 million	95	24.30%
	Above 10 million	84	21.48%
Average per transaction (RMB)	Below 100	44	11.25%
	100 to 300	130	33.25%
	300 to 500	80	20.46%
	500 to 1000	39	9.97%
	1000 to 3000	39	9.97%
	3000 to 5000	20	5.12%
	Above 5000	39	9.97%
CBEC platform	Alibaba	329	84.14%
	AliExpress	111	28.39%
	Amazon	278	71.10%
	eBay	85	21.74%
	Wish	15	3.84%
	DHgate	22	5.63%
	Made-in-China	104	26.60%
	Global Sources	41	10.49%
	Others	3	0.77%

Note: All monetary values are reported in Renminbi (RMB).

**Table 3:** Reliability and convergent validity.

Constructs	Items	VIF	Factor Loading	CA	KMO	CR	AVE
Normative mechanism	NM1	2.017	0.719	0.801	0.795	0.805	0.508
	NM2	1.781	0.702				
	NM3	1.972	0.720				
	NM4	1.922	0.710				
Supervisory mechanism	SM1	1.907	0.679	0.800	0.787	0.804	0.506
	SM2	2.008	0.727				
	SM3	1.930	0.688				
	SM4	2.171	0.749				
Reward–punishment mechanism	RP1	1.728	0.704	0.798	0.782	0.800	0.501
	RP2	1.646	0.700				
	RP3	1.872	0.716				
	RP4	1.886	0.710				
Community building	CB1	1.638	0.722	0.838	0.802	0.831	0.552
	CB2	1.652	0.732				
	CB3	1.705	0.743				
	CB4	1.770	0.773				
Perceived risk	PR1	2.034	0.683	0.871	0.817	0.873	0.633
	PR2	2.345	0.773				
	PR3	2.944	0.863				
	PR4	2.917	0.851				
Institutional distance	ID1	1.912	0.724	0.815	0.805	0.815	0.524
	ID2	1.952	0.723				
	ID3	1.982	0.736				
	ID4	1.932	0.713				
Platform trust	PT1	1.724	0.773	0.831	0.794	0.827	0.545
	PT2	1.568	0.696				
	PT3	1.595	0.731				
	PT4	1.615	0.752				

**Table 4:** Heterotrait–monotrait analysis results.

Construct	M	SD	NM	MM	RP	CC	PR	ID	PT
NM	6.055	0.643	**0.713**						
SM	5.934	0.724	0.304	**0.711**					
RP	5.873	0.673	0.275	0.301	**0.708**				
CB	5.714	0.659	0.228	0.244	0.247	**0.743**			
PR	3.041	1.077	−0.354	−0.347	−0.346	−0.360	**0.796**		
ID	5.798	0.740	0.299	0.314	0.304	0.300	−0.477	**0.724**	
PT	5.970	0.572	0.209	0.222	0.228	0.201	−0.318	0.251	**0.739**

Note: NM = normative mechanism, SM = supervisory mechanism, RP = reward–punishment mechanism, CB = community building, PR = perceived risk, ID = institutional distance, PT = platform trust. The square root of AVE (bold) is shown on the diagonal of the matrix.

**Table 5:** Analysis of model fit.

Fit Index	χ^2^/df	RMSEA	GFI	AGFI	CFI	IFI	TLI
	1.471	0.034	0.92	0.902	0.969	0.97	0.965

**Table 6:** Direct effects.

Hypothesis	Estimate	S.E.	C.R.	*p*	Result
H1a: Normative mechanism -> Perceived risk	−0.513	0.086	−5.968	0.000	Support
H1b: Supervisory mechanism -> Perceived risk	−0.163	0.081	−2.007	0.045	Support
H1c: Reward-punishment mechanism -> Perceived risk	−0.212	0.077	−2.746	0.006	Support
H2: Community building -> Perceived risk	−0.843	0.122	−6.924	0.000	Support
H3: Perceived risk -> Platform trust	−0.357	0.042	−8.587	0.000	Support

**Table 7:** Mediation effects.

Hypothesis	Effect	Estimate	SE	Bias-Corrected 95% CI		Percentile 95% CI		Result
				Lower	Upper	Lower	Upper	
H4a: Normative mechanism -> Perceived risk -> Platform trust	Indirect effect	0.038	0.024	0.003	0.103	0.001	0.096	Support (full mediation)
	Direct effect	0.154	0.080	−0.001	0.314	−0.002	0.313	
	Total effect	0.192	0.076	0.044	0.342	0.048	0.346	
H4b: Supervisory mechanism -> Perceived risk -> Platform trust	Indirect effect	0.010	0.013	−0.007	0.049	−0.012	0.041	No support
	Direct effect	0.184	0.075	0.045	0.337	0.048	0.341	
	Total effect	0.194	0.076	0.047	0.344	0.050	0.348	
H4c: Reward–punishment mechanism -> Perceived risk -> Platform trust	Indirect effect	0.012	0.012	−0.003	0.048	−0.005	0.043	No support
	Direct effect	0.229	0.072	0.096	0.379	0.096	0.379	
	Total effect	0.241	0.073	0.105	0.392	0.106	0.392	
H5: Community building -> Perceived risk -> Platform trust	Indirect effect	0.064	0.036	0.004	0.154	0.001	0.143	Support (partial mediation)
	Direct effect	0.273	0.092	0.101	0.460	0.104	0.466	
	Total effect	0.336	0.081	0.184	0.501	0.191	0.509	

**Table 8:** Moderating effects.

Hypothesis	Estimate	S.E.	C.R.	*p*	Result
H6a: Normative mechanism × Institutional distance	0.151	0.009	−16.559	0.000	Support
H6b: Supervisory mechanism × Institutional distance	0.114	0.008	−15.071	0.000	Support
H6c: Reward–punishment mechanism × Institutional distance	0.014	0.006	−2.321	0.020	Support
H7: Community building × Institutional distance	0.104	0.007	−14.13	0.000	Support

## Data Availability

The original contributions presented in the study are included in the article. Further inquiries can be directed to the corresponding author.

## References

[B1-behavsci-15-00183] Ahluwalia P., Merhi M. I. (2020). Understanding country level adoption of e-commerce: A theoretical model including technological, institutional, and cultural factors. Journal of Global Information Management.

[B2-behavsci-15-00183] Boehe D. M. (2011). Exploiting the liability of foreignness: Why do service firms exploit foreign affiliate networks at home?. Journal of International Management.

[B3-behavsci-15-00183] Brouthers K. D., Geisser K. D., Rothlauf F. (2018). Explaining the internationalization of ibusiness firms. International entrepreneurship: The pursuit of opportunities across national borders.

[B4-behavsci-15-00183] Caixe D. F., Pavan P. C. P., Maganini N. D., Sheng H. H. (2024). Foreign institutional ownership and firm value: Evidence of “locust foreign capital” in Brazil. Emerging Markets Finance and Trade.

[B5-behavsci-15-00183] Chao M. C. H., Kumar V. (2010). The impact of institutional distance on the international diversity–performance relationship. Journal of World Business.

[B6-behavsci-15-00183] Chen G., Xu J., Qi Y. (2022). Environmental (de) centralization and local environmental governance: Evidence from a natural experiment in China. China Economic Review.

[B7-behavsci-15-00183] Chua R. Y. J., Roth Y., Lemoine J. F. (2015). The impact of culture on creativity: How cultural tightness and cultural distance affect global innovation crowdsourcing work. Administrative Science Quarterly.

[B8-behavsci-15-00183] Chung H. F., Kingshott R. P., MacDonald R. V., Putranta M. P. (2021). Dynamism and B2B firm performance: The dark and bright contingent role of B2B relationships. Journal of Business Research.

[B9-behavsci-15-00183] Cravens D. W., Lassk F. G., Low G. S., Marshall G. W., Moncrief W. C. (2004). Formal and informal management control combinations in sales organizations: The impact on salesperson consequences. Journal of Business Research.

[B10-behavsci-15-00183] Cui Y., Mou J., Cohen J., Liu Y. (2019). Understanding information system success model and valence framework in sellers’ acceptance of cross-border e-commerce: A sequential multi-method approach. Electronic Commerce Research.

[B11-behavsci-15-00183] Dau L. A., Li J., Lyles M. A., Chacar A. S. (2022). Informal institutions and the international strategy of MNEs: Effects of institutional effectiveness, convergence, and distance. Journal of International Business Studies.

[B12-behavsci-15-00183] Demirbag M., Glaister K. W., Tatoglu E. (2007). Institutional and transaction cost influences on MNEs’ ownership strategies of their affiliates: Evidence from an emerging market. Journal of World Business.

[B13-behavsci-15-00183] Deng G., Zhang J., He L., Xu Y. (2023). Research on the impact of e-commerce platform’s AI resources on seller opportunism: A cultivational governance mechanism. Nankai Business Review International.

[B14-behavsci-15-00183] Dikova D., Sahib P. R., Van Witteloostuijn A. (2010). Cross-border acquisition abandonment and completion: The effect of institutional differences and organizational learning in the international business service industry, 1981–2001. Journal of International Business Studies.

[B15-behavsci-15-00183] Dong M. C., Fang Y., Straub D. W. (2017). The impact of institutional distance on the joint performance of collaborating firms: The role of adaptive interorganizational systems. Information Systems Research.

[B16-behavsci-15-00183] Donnelly R., Purkayastha S., Manolova T. S., Edelman L. F. (2024). Institutional distance, slack resources, and foreign market entry. Journal of International Business Studies.

[B17-behavsci-15-00183] Eriksson K., Strimling P., Andersson P. A., Aveyard M., Brauer M., Gritskov V., Kiyonari T., Kuhlman D. M., Maitner A. T., Manesi Z., Molho C., Peperkoorn L. S., Rizwan M., Stivers A. W., Tian Q., Van Lange P. A. M., Vartanova I., Wu J., Yamagishi T. (2017). Cultural universals and cultural differences in meta-norms about peer punishment. Management and Organization Review.

[B18-behavsci-15-00183] Faqih K. M. (2022). Internet shopping in the COVID-19 era: Investigating the role of perceived risk, anxiety, gender, culture, and trust in the consumers’ purchasing behavior from a developing country context. Technology in Society.

[B19-behavsci-15-00183] Faraj S., von Krogh G., Monteiro E., Lakhani K. R. (2016). Special section introduction—Online community as space for knowledge flows. Information Systems Research.

[B20-behavsci-15-00183] Fasli M. (2007). On agent technology for e-commerce: Trust, security and legal issues. The Knowledge Engineering Review.

[B21-behavsci-15-00183] Geldes C., Felzensztein C., Turkina E., Durand A. (2015). How does proximity affect interfirm marketing cooperation? A study of an agribusiness cluster. Journal of Business Research.

[B22-behavsci-15-00183] Ghate R., Nagendra H. (2005). Role of monitoring in institutional performance: Forest management in Maharashtra, India. Conservation and Society.

[B23-behavsci-15-00183] Grewal R., Chakravarty A., Saini A. (2010). Governance mechanisms in business-to-business electronic markets. Journal of Marketing.

[B24-behavsci-15-00183] Guo W., Straub D., Zhang P., Cai Z. (2021). How trust leads to commitment on microsourcing platforms: Unraveling the effects of governance and third-party mechanisms on triadic microsourcing relationships. MIS Quarterly.

[B25-behavsci-15-00183] Harding C., Ireland R. W. (2022). Punishment: Rhetoric, rule, and practice.

[B26-behavsci-15-00183] Høgevold N. M., Rodriguez R., Svensson G., Roberts-Lombard M. (2022). Validating the sequential logic of quality constructs in seller-customer business relationships—Antecedents, mediator and outcomes. Journal of Business-to-Business Marketing.

[B27-behavsci-15-00183] Huang M. C., Cheng H. L., Tseng C. Y. (2014). Reexamining the direct and interactive effects of governance mechanisms upon buyer–supplier cooperative performance. Industrial Marketing Management.

[B28-behavsci-15-00183] Hui X., Liu M., Chan T. (2023). Targeted incentives, broad impacts: Evidence from an e-commerce platform. Quantitative Marketing and Economics.

[B29-behavsci-15-00183] Kadi D. C. A., Amalia M. S. (2021). The influence of brand image, perception of ease and perception of risk on purchase intention through trust in shopee. Asian Journal of Management, Entrepreneurship and Social Science.

[B30-behavsci-15-00183] Kim D. J., Ferrin D. L., Rao H. R. (2008). A trust-based consumer decision-making model in electronic commerce: The role of trust, perceived risk, and their antecedents. Decision Support Systems.

[B31-behavsci-15-00183] Kumar N., Scheer L. K., Steenkamp J. B. E. (1995). The effects of perceived interdependence on dealer attitudes. Journal of Marketing Research.

[B32-behavsci-15-00183] Lacey N. (2011). Why globalisation doesn’t spell convergence: Models of institutional variation and the comparative political economy of punishment. International and comparative criminal justice and urban governance (CUP 2011).

[B33-behavsci-15-00183] Lawrence E. R., Raithatha M., Rodriguez I. (2021). The effect of cultural and institutional factors on initiation, completion, and duration of cross-border acquisitions. Journal of Corporate Finance.

[B34-behavsci-15-00183] Li D., Wei L. Q., Cao Q., Chen D. (2021). Informal institutions, entrepreneurs’ political participation, and venture internationalization. Journal of International Business Studies.

[B35-behavsci-15-00183] Li W., Sun C., Li Y., Ertz M. (2024). Effects of business to business e-commerce platform-governance mechanisms on seller firms’ performance. Research in International Business and Finance.

[B36-behavsci-15-00183] Li Z., Pénard T. (2014). The role of quantitative and qualitative network effects in B2B platform competition. Managerial and Decision Economics.

[B37-behavsci-15-00183] Liu A., Osewe M., Shi Y., Zhen X., Wu Y. (2021). Cross-border e-commerce development and challenges in China: A systematic literature review. Journal of Theoretical and Applied Electronic Commerce Research.

[B38-behavsci-15-00183] Liu Y., Gao W. (2023). Which is more effective for platform performance: Punishments or incentives?. Industrial Marketing Management.

[B39-behavsci-15-00183] Liu Y., Wan Y., Kang J. (2023). Impact of community-based governance mechanisms on transaction intention on a second-hand trading platform. Journal of Theoretical and Applied Electronic Commerce Research.

[B40-behavsci-15-00183] Lu B., Wang Z., Zhang S. (2021). Platform-based mechanisms, institutional trust, and continuous use intention: The moderating role of perceived effectiveness of sharing economy institutional mechanisms. Information & Management.

[B41-behavsci-15-00183] Ma S., Chai Y., Jia F. (2022). Mitigating transaction risk for cross-border e-commerce firms: A multiagent-based simulation study. International Business Review.

[B42-behavsci-15-00183] Mayer R. C., Davis J. H., Schoorman F. D. (1995). An integrative model of organizational trust. Academy of Management Review.

[B43-behavsci-15-00183] Minerbo C., Kleinaltenkamp M., Brito L. A. L. (2021). Unpacking value creation and capture in B2B relationships. Industrial Marketing Management.

[B44-behavsci-15-00183] Mou J., Cui Y., Kurcz K. (2020). Trust, risk and alternative website quality in B-buyer acceptance of cross-border E-commerce. Journal of Global Information Management.

[B45-behavsci-15-00183] Nguyen-Phuoc D. Q., Oviedo-Trespalacios O., Vo N. S., Le P. T., Van Nguyen T. (2021). How does perceived risk affect passenger satisfaction and loyalty towards ride-sourcing services?. Transportation Research Part D: Transport and Environment.

[B46-behavsci-15-00183] Pan L., Fu X., Li Y. (2023). SME participation in cross-border e-commerce as an entry mode to foreign markets: A driver of innovation or not?. Electronic Commerce Research.

[B47-behavsci-15-00183] Qin L., Qu Q., Zhang L., Wu H. (2022). Platform trust in C2C e-commerce platform: The sellers’ cultural perspective. Information Technology and Management.

[B48-behavsci-15-00183] Rodrigues A. R. D., Ferreira F. A., Teixeira F. J., Zopounidis C. (2022). Artificial intelligence, digital transformation and cybersecurity in the banking sector: A multi-stakeholder cognition-driven framework. Research in International Business and Finance.

[B49-behavsci-15-00183] Sahasranamam S., Nandakumar M. K. (2020). Individual capital and social entrepreneurship: Role of formal institutions. Journal of Business Research.

[B50-behavsci-15-00183] San-Martín S., Jimenez N. (2017). Curbing electronic shopper perceived opportunism and encouraging trust. Industrial Management & Data Systems.

[B51-behavsci-15-00183] Shen H., Yu J., Zhang H., Gou J., Zhang X. (2022). How does social support affect the retention willingness of cross-border e-commerce sellers?. Frontiers in Psychology.

[B52-behavsci-15-00183] Simonin B. L. (1997). The importance of collaborative know-how: An empirical test of the learning organization. The Academy of Management Journal.

[B53-behavsci-15-00183] Wang D. T., Gu F. F., Dong M. C. (2013). Observer effects of punishment in a distribution network. Journal of Marketing Research.

[B54-behavsci-15-00183] Wang J., Cai S., Xie Q., Chen L. (2022). The influence of community engagement on seller opportunistic behaviors in e-commerce platform. Electronic Commerce Research.

[B55-behavsci-15-00183] Wang R., Sui Y. (2019). Political institutions and foreign banks’ risk-taking in emerging markets. Journal of Multinational Financial Management.

[B56-behavsci-15-00183] Wei K., Li Y., Zha Y., Ma J. (2019). Trust, risk and transaction intention in consumer-to-consumer e-marketplaces: An empirical comparison between buyers’ and sellers’ perspectives. Industrial Management & Data Systems.

[B57-behavsci-15-00183] Yamamoto H., Ohshima H. (2017). Proactive or reactive? Platform governance strategy in C2C marketplace. Twenty First Pacific Asia Conference on Information Systems.

[B58-behavsci-15-00183] Yang Q., Pang C., Liu L., Yen D. C., Tarn J. M. (2015). Exploring consumer perceived risk and trust for online payments: An empirical study in China’s younger generation. Computers in Human Behavior.

[B59-behavsci-15-00183] Yan Z., Lu X., Chen Y., Wang K. (2023). Institutional distance, internationalization speed and cross-border e-commerce platform utilization. Management Decision.

[B60-behavsci-15-00183] Zhang L., Jin Y., Xia L., Xu B., Abdullah S. M. S. (2022). The effects of social distance and asymmetric reward and punishment on individual cooperative behavior in dilemma situations. Frontiers in Psychology.

[B61-behavsci-15-00183] Zhang Z., Zhang C., Shen L. (2020). Deterring dealer slackness: The role of supplier incentives and monitoring and the market environment. Journal of Business Research.

[B62-behavsci-15-00183] Zheng H., Xu B., Lin Z. (2019). Seller’s creditworthiness in the online service market: A study from the control perspective. Decision Support Systems.

[B63-behavsci-15-00183] Zucker L. G. (1986). Production of trust: Institutional sources of economic structure, 1840–1920. Research in Organizational Behavior.

